# Disparities in the prevalence of clinical features between systemic juvenile idiopathic arthritis and adult-onset Still’s disease

**DOI:** 10.1093/rheumatology/keac027

**Published:** 2022-01-25

**Authors:** Piero Ruscitti, Valentina Natoli, Alessandro Consolaro, Roberta Caorsi, Silvia Rosina, Gabriella Giancane, Roberta Naddei, Ilenia Di Cola, Claudia Di Muzio, Onorina Berardicurti, Daniela Iacono, Ilenia Pantano, Gelsomina Rozza, Silvia Rossi, Ludovico De Stefano, Silvia Balduzzi, Antonio Vitale, Francesco Caso, Luisa Costa, Marcella Prete, Luca Navarini, Annamaria Iagnocco, Fabiola Atzeni, Giuliana Guggino, Federico Perosa, Luca Cantarini, Bruno Frediani, Carlomaurizio Montecucco, Francesco Ciccia, Paola Cipriani, Marco Gattorno, Roberto Giacomelli, Angelo Ravelli

**Affiliations:** Department of Biotechnological and Applied Clinical Sciences, University of L'Aquila, L'Aquila; Dipartimento di Neuroscienze, Riabilitazione, Oftalmologia, Genetica e Scienze Materno-Infantili, Università degli Studi di Genova, Genoa; Dipartimento di Neuroscienze, Riabilitazione, Oftalmologia, Genetica e Scienze Materno-Infantili, Università degli Studi di Genova, Genoa; UOC Clinica Pediatrica e Reumatologia, IRCCS Istituto Giannina Gaslini, Genoa; UOC Clinica Pediatrica e Reumatologia, IRCCS Istituto Giannina Gaslini, Genoa; UOC Clinica Pediatrica e Reumatologia, IRCCS Istituto Giannina Gaslini, Genoa; Dipartimento di Neuroscienze, Riabilitazione, Oftalmologia, Genetica e Scienze Materno-Infantili, Università degli Studi di Genova, Genoa; UOC Clinica Pediatrica e Reumatologia, IRCCS Istituto Giannina Gaslini, Genoa; UOC Clinica Pediatrica e Reumatologia, IRCCS Istituto Giannina Gaslini, Genoa; Department of Translational Medical Sciences, Section of Pediatrics, University of Naples Federico II; Department of Biotechnological and Applied Clinical Sciences, University of L'Aquila, L'Aquila; Department of Biotechnological and Applied Clinical Sciences, University of L'Aquila, L'Aquila; Department of Biotechnological and Applied Clinical Sciences, University of L'Aquila, L'Aquila; Department of Precision Medicine, University of Campania ‘Luigi Vanvitelli’, Naples; Department of Precision Medicine, University of Campania ‘Luigi Vanvitelli’, Naples; Department of Precision Medicine, University of Campania ‘Luigi Vanvitelli’, Naples; Rheumatology Department, Fondazione IRCCS Policlinico San Matteo, University of Pavia, Pavia; Rheumatology Department, Fondazione IRCCS Policlinico San Matteo, University of Pavia, Pavia; Rheumatology Department, Fondazione IRCCS Policlinico San Matteo, University of Pavia, Pavia; Research Center of Systemic Auto Inflammatory Diseases, Behçet's Disease and Rheumatology-Ophthalmology Collaborative Uveitis Center, Department of Medical Sciences, Surgery and Neurosciences, Rheumatology Unit, Policlinico ‘Le Scotte’, University of Siena, Siena; Rheumatology Unit, Department of Clinical Medicine and Surgery, University of Naples Federico II, Naples; Rheumatology Unit, Department of Clinical Medicine and Surgery, University of Naples Federico II, Naples; Rheumatic and Systemic Autoimmune Diseases Unit, Department of Biomedical Science and Human Oncology (DIMO), University of Bari Medical School, Bari; Rheumatology and Immunology Unit, Department of Medicine, University of Rome Campus Biomedico, Rome; Academic Rheumatology Centre, Ospedale Mauriziano – Dipartimento Scienze Cliniche e Biologiche, Università degli Studi di Torino, Turin; Rheumatology Unit, Department of Clinical and Experimental Medicine, University of Messina, Messina; Rheumatology Section, Department of Health Promotion, Mother and Child Care, Internal Medicine and Medical Specialties, University Hospital ‘P. Giaccone’, Palermo; Rheumatic and Systemic Autoimmune Diseases Unit, Department of Biomedical Science and Human Oncology (DIMO), University of Bari Medical School, Bari; Research Center of Systemic Auto Inflammatory Diseases, Behçet's Disease and Rheumatology-Ophthalmology Collaborative Uveitis Center, Department of Medical Sciences, Surgery and Neurosciences, Rheumatology Unit, Policlinico ‘Le Scotte’, University of Siena, Siena; Research Center of Systemic Auto Inflammatory Diseases, Behçet's Disease and Rheumatology-Ophthalmology Collaborative Uveitis Center, Department of Medical Sciences, Surgery and Neurosciences, Rheumatology Unit, Policlinico ‘Le Scotte’, University of Siena, Siena; Rheumatology Department, Fondazione IRCCS Policlinico San Matteo, University of Pavia, Pavia; Department of Precision Medicine, University of Campania ‘Luigi Vanvitelli’, Naples; Department of Biotechnological and Applied Clinical Sciences, University of L'Aquila, L'Aquila; UOC Clinica Pediatrica e Reumatologia, IRCCS Istituto Giannina Gaslini, Genoa; Rheumatology and Immunology Unit, Department of Medicine, University of Rome Campus Biomedico, Rome; Dipartimento di Neuroscienze, Riabilitazione, Oftalmologia, Genetica e Scienze Materno-Infantili, Università degli Studi di Genova, Genoa; Direzione Scientifica, IRCCS Istituto Giannina Gaslini, Genoa, Italy

**Keywords:** systemic juvenile idiopathic arthritis, adult-onset Still’s disease

## Abstract

**Objective:**

To compare clinical features and treatments of patients with systemic JIA (sIJA) and adult-onset Still’s disease (AOSD).

**Methods:**

The clinical charts of consecutive patients with sJIA by International League of Association of Rheumatology criteria or AOSD by Yamaguchi criteria were reviewed. Patients were seen at a large paediatric rheumatology referral centre or at 10 adult rheumatology academic centres. Data collected included clinical manifestations, inflammation biomarkers, systemic score, macrophage activation syndrome (MAS), parenchymal lung disease, disease course, disability, death and medications administered.

**Results:**

A total of 166 patients (median age at diagnosis 5 years) with sJIA and 194 patients with AOSD (median age at diagnosis 41 years) were included. The frequency of fever, rash, arthralgia, abdominal pain, MAS, parenchymal lung disease and increased acute phase reactants and ferritin were comparable between the two cohorts. Patients with sJIA had a higher prevalence of arthritis, whereas patients with AOSD had experienced leucocytosis and extra-articular organ involvement more frequently. Patients with AOSD were given more commonly low-dose corticosteroids, whereas biologic DMARDs were administered first-line more frequently in patients with sJIA.

**Conclusion:**

We found remarkable disparities in the prevalence of clinical manifestations between the two illnesses, which may partly depend on their classification by different criteria.

Rheumatology key messages

- A remarkable disparity in prevalence of several clinical manifestations between sJIA and AOSD was observed.
- Patients with sJIA and AOSD had a higher frequency of arthritis and extra-articular manifestations, respectively.
- There were differences in the use of medications between pediatric and adult rheumatologists.

## Introduction

Still’s disease is an uncommon inflammatory disorder that can affect both children and adults and is characterized by the triad of daily spiking fever, arthritis and evanescent salmon-coloured skin rash [1, 2]. This disorder was first described in children by George F. Still in 1897 [3], whereas the adult form was defined in 1971 by Eric Bywaters [4]. Currently, this condition is named systemic JIA (sJIA) in children and adult-onset Still’s disease (AOSD) in adults [1, 2].

A large body of evidence supports the similarity between AOSD and sJIA. Beside the above-mentioned cardinal features, the two illnesses share many other clinical manifestations, including hepatomegaly, splenomegaly, lymphadenopathy and serositis. Furthermore, they exhibit common laboratory abnormalities, including increased white blood cell count, ESR, CRP and hyperferritinemia. In addition, the disease course and prognosis are comparable [5, 6]. For both sJIA and AOSD, a phenotypic dichotomy has been recognized, with a more systemic inflammatory phenotype and a more articular chronic phenotype. Additional clinical similarities include a distinctive predisposition to develop life-threatening complications, such as macrophage activation syndrome (MAS) and interstitial lung disease [7, 8].

Biologic data suggests that AOSD and SJIA are also very similar in terms of pathophysiology, as the innate immune system plays a prominent role in both conditions [9–11]. The overexpression of inflammatory cytokines, such as IL-1, IL-6, IL-18 and calcium binding proteins, as well as the striking response to IL-1 and IL-6 inhibition, have led to postulating that they should be considered complex, polygenic autoinflammatory syndromes, rather than autoimmune diseases [9–11]. The finding of similar associations with HLA alleles and cytokine gene polymorphisms indicates that sJIA and AOSD may be indistinguishable on a molecular level [12].

Based on the compelling evidence of their similarity, most experts believe that sJIA and AOSD are the same disease occurring in different age groups [13]. However, the terminology remains different and diverse classification criteria are used. This discordance is partly explained by the scarcity of published data on the comparison of the two disorders, which mostly come from isolated case reports or small patient series. The lack of information may also depend on sJIA patients being seen by paediatric rheumatologists and those with AOSD by adult rheumatologists.

Against this background, the purpose of the present study was to compare the features of a large sample of patients with sJIA and AOSD seen in paediatric and adult rheumatology settings, respectively.

## Methods

### Study design and patient selection

Patient data were collected through the review of clinical charts. To be included, patients should meet the ILAR criteria for sJIA [14] or the Yamaguchi criteria for AOSD [15] and have a follow-up ≥6 months after disease onset. Patients with sJIA who at disease onset had the classic extra-articular manifestations of sJIA, but did not meet the ILAR criteria because of the absence of arthritis, were classified as sJIA by such criteria if they had developed arthritis during the disease course. Patients with sJIA were seen consecutively at the Giannina Gaslini Institute of Genoa, Italy, a paediatric rheumatology referral centre whose catchment area extends to the entire country. Consecutive patients with AOSD were enrolled by the GIRRCS (Gruppo Italiano di Ricerca in Reumatologia Clinica e Sperimentale), a collaborative study group of academic Italian adult rheumatologists. Patients were seen between January 2001 and June 2021.

### Data collection

Demographic data, clinical features, inflammation biomarkers and systemic score were registered at diagnosis, before the start of immunosuppressive therapies. The systemic score assigns one point to each of the following 12 manifestations: fever, typical rash, pleuritis, pneumonia, pericarditis, hepatomegaly or abnormal liver function tests, splenomegaly, lymphadenopathy, leucocytosis >15* *000/mm^3^, sore throat, myalgia and abdominal pain (maximum score: 12 points) [16]. The occurrence of MAS, parenchymal lung disease [17], comorbidities and death, and medication administered were recorded by reviewing patient history. Based on disease course until last visit, patients were stratified into three patterns: monocyclic, polycyclic and chronic continuous. Monocyclic course was defined as a single episode lasting >2 months but <1 year, followed by sustained remission through the whole follow-up; polycyclic course was defined as recurrent systemic flares with remission between flares; chronic continuous course was defined as persistence of symptoms throughout the whole follow-up or the need for chronic therapy.

First-line biologic DMARDs were administered after failure of glucocorticoids and/or synthetic DMARDs, second-line biologic DMARDs were administered after failure of first-line biologic DMARDs and third-line biologic DMARDs were administered after failure of second-line biologic DMARDs.

Study data were collected through a standardized case report form and entered in an Excel spreadsheet.

### Ethics

The ethics committee of Azienda Sanitaria Locale 1 Avezzano-Sulmona-L’Aquila, L'Aquila, Italy (No. 0139815/16) approved the study, which was performed according to the Good Clinical Practice guidelines and the Declaration of Helsinki. After approval of the ethics committee, we collected written informed consents for patients presently and actively followed-up in each centre. However, owing to the retrospective nature of the study, for those patients who were no longer followed-up (lost to follow-up or died during the time-period of assessment), after having made every reasonable effort to contact them, we used the fully anonymized clinical data according to the Italian Law on privacy only for research purposes without any other intended aim [Garante per la protezione dei dati personali, Autorizzazione n. 9/2016—Autorizzazione generale al trattamento dei dati personali effettuato per scopi di ricerca scientifica—15 December 2016 (5805552)].

### Statistics

Comparisons of quantitative variables between the two groups were made by means of Mann–Whitney *U* test. Categorical data were compared by *χ*^2^ test, or by Fisher’s exact test in case of expected frequencies <5. *P*-values <0.05 were considered statistically significant.

## Results

A total of 166 patients with sJIA (median age at diagnosis 5 years) and 194 patients with AOSD (median age at diagnosis 41 years) were included in the study. The proportion of patients diagnosed in the date ranges 2001–2005, 2006–2010, 2011–2015 and after 2016 was 1.8%, 13.8%, 25.3% and 59.1%, respectively, for sJIA and 2.6%, 7.7%, 17.5% and 72.2%, respectively, for AOSD (*P* = 0.038). The comparison of clinical features between the two samples is presented in Table 1. Sex ratio and duration of follow-up as well as frequency of fever, rash, arthralgia, abdominal pain, MAS, parenchymal lung disease, increased ESR, CRP and ferritin were comparable between the two groups.

**Table 1:** Comparison of clinical features between sJIA and AOSD patients

Feature	sJIA(*n* = 166)	AOSD(*n* = 194)	*P*-value
Male	79 (47.9)	102 (52.6)	0.40
Median (IQR) duration of follow-up, years	5.0 (8.6)	4.0 (6.1)	0.31
Fever^a^	166 (100.0)	191 (98.5)	0.63
Musculoskeletal features^a^			
Myalgia	22 (13.3)	117 (60.3)	<0.0001
Arthralgia	146 (88.0)	162 (84.0)	0.31
Arthritis	146^b^ (88.0)	116 (59.8)	<0.0001
Erosive arthritis	32 (19.3)	15 (7.7)	0.002
Joint replacement^c^	11 (6.6)	4 (2.1)	0.037
Organ involvement^a^			
Skin rash	122 (73.5)	142 (73.2)	0.99
Sore throat	17 (10.2)	115 (59.3)	<0.0001
Liver involvement	43 (25.9)	110 (56.7)	<0.0001
Lymphadenopathy	27 (16.3)	101 (52.1)	<0.0001
Splenomegaly	26 (15.7)	89 (45.9)	<0.0001
Pericarditis	12 (7.2)	40 (20.6)	<0.0001
Pleuritis	6 (3.6)	37 (19.1)	<0.0001
Abdominal pain	12 (7.2)	18 (9.3)	0.57
Median (IQR) systemic score^e^^,f^	3 (2–5)	5 (4–7)	<0.0001
Laboratory abnormalities^a^			
Leucocyte count >15* *000/mm^3^	59 (35.5)	122 (62.9)	<0.0001
CRP >0.5 mg/dl	97/98 (99.0)	174/185 (94.1)	0.063
ESR >20, mm/h	90/95 (94.7)	166/181 (91.7)	0.467
Median (IQR) ferritin, ng/ml^d^	1074.0 (325–33 700)	1105.0 (3–150 000)	0.57
Complications^c^			
Macrophage activation syndrome	14 (8.4)	23 (11.9)	0.30
Parenchymal lung disease	7 (4.2)	18 (9.3)	0.06
Disability^g^			
No disability	112 (67.5)	158 (81.4)	0.003
Mild disability	36 (21.7)	25 (12.9)	0.03
Moderate disability	12 (7.2)	9 (4.6)	0.37
Severe disability	5 (3.0)	0 (0.0)	0.02
Disease patterns^g^			
Monocyclic	41 (24.7)	67 (34.5)	0.055
Polycyclic	75 (42.2)	87 (44.8)	0.92
Chronic	36 (21.7)	27 (13.9)	0.07
Mortality^g^	0 (0.0)	12 (6.2)	0.001
Comorbidities	41 (24.7)	108 (55.7)	<0.0001

Data are the number (percentage) unless otherwise indicated.

a Collected at the time of diagnosis.

b 20 patients developed arthritis during follow-up.

c Collected at the time of diagnosis and/or during the follow-up.

d Available in 95 sJIA patients and 158 AOSD patients.

e Available in 146 sJIA patients and 194 AOSD patients.

f The systemic score assigns one point to each of the following 12 manifestations: fever, typical rash, pleuritis, pneumonia, pericarditis, hepatomegaly or abnormal liver function tests, splenomegaly, lymphadenopathy, leucocytosis >15* *000/mm^3^, sore throat, myalgia and abdominal pain (maximum score: 12 points).

g Collected at the end of follow-up.

AOSD: adult-onset Still’s disease; IQR: interquartile range; sJIA: systemic JIA.

Patients with sJIA had a higher prevalence of arthritis (which in 20 patients was not present at onset, but developed during disease course) and erosive arthritis, and had undergone more frequently joint replacement surgery than patients with AOSD. Patients with AOSD had experienced more frequently myalgia, sore throat, hepatomegaly, splenomegaly, lymphadenopathy, pericarditis, pleuritis and leucocytosis, and had a greater systemic score than patients with sJIA.

The proportion of the three course patterns was comparable across the two samples, whereas the frequency of comorbidities and mortality was higher in AOSD patients. Comorbidities, defined as coexisting medical conditions distinct from the principal diagnosis, recorded in the two cohorts are listed in detail in Supplementary Table S1, available at *Rheumatology* online. Mortality was seen only in patients with AOSD and was attributable to MAS or parenchymal lung disease.

The comparison of medications administered during the disease course is shown in Table 2. Glucocorticoids were given more commonly to patients with AOSD, although the difference was significant only for the low-dose category. The disparity in the frequency of usage of glucocorticoids might not be explained by AOSD patients being diagnosed at earlier date ranges as more patients with AOSD than with sJIA were diagnosed after 2016 (see above). Prescription of synthetic DMARDs was more frequent among patients with AOSD. MTX was given with equal frequency in the two populations, whereas ciclosporin and HCQ were selected more commonly in sJIA and AOSD, respectively.

**Table 2:** Comparison of drug therapies between SJIA and AOSD patients

	sJIA(*n* = 166)	AOSD(*n* = 194)	*P*-value
Glucocorticoids^a^	141 (84.9)	180 (92.7)	0.026
Low dose	65 (39.2)	100 (51.5)	0.020
High dose	60 (36.1)	80 (41.2)	0.28
Synthetic DMARDs	56 (33.7)	123 (63.4)	<0.0001
MTX	39 (69.6)	89 (72.4)	0.71
Ciclosporin	17 (30.4)	18 (14.6)	0.014
HCQ	1 (1.8)	17 (13.8)	0.013
SSZ	0 (0)	4 (3.3)	0.30
First-line biologic DMARDs	116 (69.9)	79 (40.7)	<0.0001
IL-1 inhibitors	66 (56.9)	48 (60.8)	0.59
IL-6 inhibitors	3 (2.6)	14 (17.7)	<0.0001
TNF inhibitors	48 (41.4)	13 (16.5)	<0.0001
Second-line biologic DMARDs	63 (38.0)	26 (13.4)	<0.0001
IL-1 inhibitors	31 (49.2)	12 (46.2)	0.79
IL-6 inhibitors	9 (14.3)	8 (30.8)	0.07
TNF inhibitors	22 (34.9)	5 (19.2)	0.14
Third-line biologic DMARDs	32 (19.3)	4 (2.1)	<0.0001
IL-1 inhibitors	12 (37.5)	3 (75.0)	0.15
IL-6 inhibitors	10 (31.3)	1 (25.0)	0.79
TNF inhibitors	10 (31.3)	1 (25.0)	0.79

Data are the number (percentage).

a Glucocorticoids were codified in the categories high dosage and low dosage based on the treatment regimen administered for the longest time period: (i) low/medium dose: ≤0.5 mg/kg/day of prednisone; (ii) high dose: >0.5 mg/kg/day of prednisone.

AOSD: adult-onset Still’s disease; sJIA: systemic JIA.

Biologic DMARDs were administered as first-line DMARD more frequently in patients with sJIA than in those with AOSD. IL-1 inhibitors were used with equal frequency in the two cohorts, whereas IL-6 blockers and TNF antagonists were chosen more commonly in AOSD and sJIA, respectively. Notably, before the approval of IL-6 blockers, TNF antagonists were administered more frequently in patients with sJIA and, to a lesser extent, in patients with AOSD (see Supplementary Table S2, available at *Rheumatology* online).

The biologic DMARD most frequently administered was anakinra (54.3% and 44.3% of patients with sJIA and AOSD, respectively). The second and third were etanercept (37.9%) and tocilizumab (2.6%) in sJIA, and tocilizumab (17.7%) and canakinumab (15.2%) in AOSD.

## Discussion

Although our results confirmed the basic similarities of sJIA and AOSD, they disclosed a remarkable disparity in the prevalence of several clinical manifestations. The most relevant difference regarded a higher frequency of arthritis in sJIA patients and a greater prevalence of involvement of extra-articular organs and leucocytosis in the AOSD population. A substantial selection bias is unlikely as the two cohorts were composed of consecutive patients seen in a large paediatric rheumatology referral centre or in 10 primary academic centres of adult rheumatology.

The observed disparities could be explained by differences in the underlying disease process, with sJIA being more prone to the development of joint disease and AOSD having a greater tendency to fuel systemic inflammation. However, the diverse criteria used to classify the two disorders could also play a role. In the current ILAR criteria for sJIA, the presence of arthritis at presentation is mandatory, whereas the Yamaguchi criteria used for AOSD only require the presence of arthralgia for more than two weeks. Thus, because all sJIA patients had to meet the ILAR criteria, this implies that only sJIA patients with arthritis have been included. Hence, the difference in prevalence of arthritis between AOSD and sJIA could be an expected consequence. Note that 20 sJIA patients who developed arthritis later on the disease course were included as well.

Because arthritis in sJIA can appear at any time over the disease course, sometimes years after the onset of systemic manifestations, and there are also patients who possess the same clinical and biological systemic features observed in sJIA but never develop arthritis, it has been argued that the absence of arthritis should not exclude the diagnosis [18]. In line with this contention, a recent proposal for a new classification for JIA considered sJIA as equivalent to AOSD and choose a modified version of the Yamaguchi criteria, with minor modifications. By these criteria, the presence of arthritis is no longer required, and in addition to fever, the presence of classic skin rash plus at least two minor criteria is sufficient for the diagnosis [19].

Other differences between the study populations involved the use of medications. The more common prescription of ciclosporin by paediatric rheumatologists and of low-dose corticosteroids and HCQ by adult rheumatologists may reflect diversities in practice. Although IL-1 inhibitors were selected most commonly and administered with equal frequency, a relative preference for IL-6 blockers by adult rheumatologists and for TNF inhibitors by paediatric rheumatologists was observed.

Our study is not without limitations. Patient data were collected through the retrospective review of clinical charts. A retrospective analysis is subject to missing and possibly erroneous data. Because the study patients were seen over a wide time period (2001–2021), some kind of selection bias cannot be excluded. During the timeframe of patient inclusion, there were major variations in the treatment approach, which might have affected the study figures. Our effort did not take into account the recent scientific evidence for biomarkers of immune activation and systemic inflammation, which may open the way to a new molecular nomenclature [20].

In conclusion, although our results support the hypothesis of sJIA and AOSD being a continuum of the same disease, we found remarkable disparities in the prevalence of some clinical manifestations between the two illnesses. Therapeutic approaches were also partly different. Large prospective cohort studies incorporating the newly proposed criteria for sJIA are needed to gain further insights into the relationship between the two conditions. The research agenda also calls for consensus efforts among paediatric and adult rheumatologists aimed to harmonize nomenclature, classification and treatment protocols.

## Acknowledgements

We confirm that all of the authors meet all of the criteria for authorship in the ICMJE Recommendations because all contributed to the conception or design of the work, acquisition and interpretation of data. All authors critically reviewed the manuscript and approved the final version.

*Funding:* No specific funding was received from any bodies in the public, commercial or not-for-profit sectors to carry out the work described in this article.

*Disclosure statement:* The authors have declared no conflicts of interest.

## Supplementary Material

keac027_Supplementary_Data

## Data Availability

All data generated by the research are available upon request to the authors.
