# Supplementary material for: Disparities in the prevalence of clinical features between systemic juvenile idiopathic arthritis and adult-onset Still’s disease
Source: Rheumatology (Oxford). 2022 Jan 25;61(10):4124–9. doi: 10.1093/rheumatology/keac027 (PMC9536787; doi:10.1093/rheumatology/keac027)
Supplement: keac027_Supplementary_Data [file keac027_supplementary_data.docx]

Supplementary Table S1. Comorbidities in patients with sJIA and with AOSD

| sJIA  (n = 166) | | AOSD  (n = 194) | |
| --- | --- | --- | --- |
|  | N (%) |  | N (%) |
| Any comorbidity | 41 (24.7) | Any comorbidity | 108 (55.7) |
| Cardiovascular malformations | 7 (4.2) | High blood pressure | 27 (25.0) |
| Heart valve disease | 5 (3.0) | Thyroid disease | 16 (14.8) |
| Neurological disorders | 3 (1.8) | Gastrointestinal disorders | 15 (13.8) |
| Obesity | 3 (1.8) | Neoplasm | 10 (9.3) |
| Psoriasis | 2 (1.2) | Dyslipidaemia | 10 (9.3) |
| Fibromyalgia | 2 (1.2) | Type 2 Diabetes | 9 (8.3) |
| Hypertension | 2 (1.2) | Fibromyalgia | 7 (6.5) |
| Anxiety | 2 (1.2) | Cardiovascular disease | 5 (4.6) |
| Thyroid disease | 2 (1.2) | Depression | 4 (3.7) |
| Bronchial asthma | 2 (1.2) | Chronic obstructive pulmonary disease | 4 (3.7) |
| Vesicoureteral reflux | 2 (1.2) | Psoriasis | 3 (2.7) |
| Mediterranean anaemia | 1 (0.6) | Osteopenia | 2 (1.9) |
| Bilateral hypoacusia | 1 (0.6) | Inguinal hernia | 2 (1.9) |
| Preputial phimosis | 1(0.6) | HBV infection | 2 (1.9) |
| Deficit of IgA | 1 (0.6) | Iron deficiency anaemia | 2 (1.9) |
| Camptodactyly | 1 (0.6) | Prostatic hypertrophy | 2 (1.9) |
| Ewing sarcoma | 1 (0.6) | Hyperhomocysteinemia | 1 (0.9) |
| Cutaneous xerosis | 1 (0.6) | Neurosensorial hypoacusia | 1 (0.9) |
| HBV infection | 1 (0.6) | Atopic dermatitis | 1 (0.9) |
| Thalassemia major | 1 (0.6) | Hepatic focal nodular hyperplasia | 1 (0.9) |
| Oesophageal atresia and trachea-oesophageal fistula | 1 (0.6) | Osteoporosis | 1 (0.9) |
| Atopic Dermatitis | 1 (0.6) | Bronchial asthma | 1 (0.9) |
| Enuresis | 1 (0.6) | Endometriosis | 1 (0.9) |
| Adolescent idiopathic scoliosis | 1 (0.6) | Hyperuricemia | 1 (0.9) |
| Allergic conjunctivitis | 1 (0.6) | Transitory ischemic attack | 1 (0.9) |
| Allergic rhinitis | 1 (0.6) | HCV infection | 1 (0.9) |
| Haemolytic uremic syndrome | 1 (0.6) | Epilepsy | 1 (0.9) |
| Pauci-immune nephropathy | 1 (0.6) | Turner Syndrome | 1 (0.9) |
| Non-ossifying fibroma | 1 (0.6) | Latent tuberculosis | 1 (0.9) |
|  |  | Myelodysplastic syndrome | 1 (0.9) |

**Supplementary Table S2. Comparison of therapeutic strategies before and after the approval of IL-6 inhibitors in sJIA and AOSD.**

|  | sJIA  Pre-approval of IL-6 inhibitors^a^  (n=42) | sJIA  Post-approval of  IL-6 inhibitors^b^ (n = 124) | P values  a vs b | AOSD  Pre-approval of  IL-6 inhibitors^c^  (n = 28) | AOSD  Post-approval of  IL-6 inhibitors^d^  (n = 166) | P values  c vs d | Total  P values |
| --- | --- | --- | --- | --- | --- | --- | --- |
| Glucocorticoids^§^ | 34 (80.9) | 107 (86.3) | 0.455 | 26 (92.8) | 154 (92.8) | 0.999 | 0.290 |
| Low dose | 15 (35.7) | 50 (40.3) | 0.715 | 16 (57.1) | 84 (50.6) | 0.547 | 0.791 |
| High dose | 13 (30.9) | 47 (37.9) | 0.462 | 11 (39.3) | 71 (42.3) | 0.837 | 0.344 |
| Synthetic DMARDs | 18 (42.8) | 38 (30.6) | 0.186 | 20 (71.4) | 103 (62.0) | 0.401 | 0.677 |
| Methotrexate | 14 (33.3) | 25 (20.2) | 0.140 | 13 (46.4) | 76 (45.8) | 0.999 | 0.346 |
| Cyclosporine | 5 (11.9) | 12 (9.6) | 0.769 | 5 (17.8) | 13 (7.8) | 0.148 | 0.176 |
| Hydroxychloroquine | 0 (0.0) | 1 (0.8) | 0.999 | 6 (21.4) | 11 (6.6) | **0.021** | 0.132 |
| Sulfasalazine | 0 (0.0) | 0 (0.0) | 0.999 | 0 (0.0) | 4 (2.4) | 0.999 | 0.999 |
| First-line biologic DMARDs | 34 (80.9) | 79 (63.7) | 0.055 | 9 (32.4) | 56 (33.7) | 0.679 | 0.082 |
| IL-1 inhibitors | 13 (30.9) | 53 (42.7) | 0.203 | 4 (14.3) | 44 (26.5) | 0.236 | **0.038** |
| TNF inhibitors | 21 (50.0) | 27 (21.8) | **0.001** | 4 (14.3) | 9 (5.4) | 0.098 | **<0.0001** |
| Second-line biologic DMARDs | 24 (57.1) | 30 (24.2) | **0.001** | 3 (10.7) | 22 (9.0) | 0.773 | **<0.0001** |
| IL-1 inhibitors | 12 (28.5) | 19 (15.3) | 0.070 | 2 (7.1) | 10 (6.0) | 0.685 | **0.038** |
| TNF inhibitors | 12 (28.6) | 10 (8.1) | **0.003** | 1 (3.6) | 4 (2.4) | 0.545 | **<0.0001** |
| Third-line biologic DMARDs | 8 (19.0) | 19 (11.3) | **0.040** | 0 (0.0) | 3 (1.8) | 0.999 | **0.013** |
| IL-1 inhibitors | 6 (14.3) | 6 (4.8) | 0.776 | 0 (0.0) | 3 (1.8) | 0.625 | 0.087 |
| TNF inhibitors | 2 (4.8) | 8 (6.4) | 0.999 | 0 (0.0) | 1 (0.6) | 0.999 | 0.999 |

Data are the number (percentage)

sJIA = systemic juvenile idiopathic arthritis; AOSD: adult-onset Still’s disease; DMARDs = disease-modifying antirheumatic drugs

^§^Glucocorticoids were categorized in the categories of high and low dosage based on the regimen administered for the longest time period: i. low/medium dose = ≤ 0.5 mg/kg/day of prednisone; ii. high dose = > 0.5 mg/kg/day of prednisone.
